# Sub-10 nm nanogap fabrication on suspended glassy carbon nanofibers

**DOI:** 10.1038/s41378-019-0120-z

**Published:** 2020-01-27

**Authors:** Arnoldo Salazar, Samira Hosseini, Margarita Sanchez-Domínguez, Marc. J. Madou, Alejandro Montesinos-Castellanos, Sergio O. Martinez-Chapa

**Affiliations:** 1 School of Engineering and Sciences, Tecnológico de Monterrey, Eugenio Garza Sada 2501, Monterrey, NL 64849 México; 2 Centro de Investigación en Materiales Avanzados, S. C. (CIMAV), Unidad Monterrey Parque de Investigación e Innovación Tecnológica, Apodaca, NL 66628 México; 3 Department of Mechanical and Aerospace Engineering, University of California Irvine, Engineering Gateway 4200, Irvine, CA 92697 USA

**Keywords:** Nanoscale devices, Nanowires

## Abstract

Glassy carbon nanofibers (GCNFs) are considered promising candidates for the fabrication of nanosensors for biosensing applications. Importantly, in part due to their great stability, carbon electrodes with sub-10 nm nanogaps represent an attractive platform for probing the electrical characteristics of molecules. The fabrication of sub-10 nm nanogap electrodes in these GCNFs, which is achieved by electrically stimulating the fibers until they break, was previously found to require fibers shorter than 2 µm; however, this process is generally hampered by the limitations inherent to photolithographic methods. In this work, to obtain nanogaps on the order of 10 nm without the need for sub-2 µm GCNFs, we employed a fabrication strategy in which the fibers were gradually thinned down by continuously monitoring the changes in the electrical resistance of the fiber and adjusting the applied voltage accordingly. To further reduce the nanogap size, we studied the mechanism behind the thinning and eventual breakdown of the suspended GCNFs by controlling the environmental conditions and pressure during the experiment. Following this approach, which includes performing the experiments in a high-vacuum chamber after a series of carbon dioxide (CO_2_) purging cycles, nanogaps on the order of 10 nm were produced in suspended GCNFs 52 µm in length, much longer than the ~2 µm GCNFs needed to produce such small gaps without the procedure employed in this work. Furthermore, the electrodes showed no apparent change in their shape or nanogap width after being stored at room temperature for approximately 6 months.

## Extending the reach of carbon electrodes

A process for the controlled fabrication of glassy carbon nanofiber (GCNF) electrodes could lead to molecular sensors with lower cost and improved durability. GCNFs are a promising alternative to precious metals for the manufacture of nanogap electrodes, but existing manufacturing methods require ultra-short fibers, which are difficult to produce and manipulate. Researchers led by Sergio O. Martinez-Chapa of Tecnológico de Monterrey in Mexico have now devised a strategy for continuously monitoring the structural integrity of GCNF fibers as they are thinned down during the electrode fabrication process. This allowed their team to work with fibers that are far longer—52 μm in length rather than 2 μm—while still achieving the extremely narrow separation required for nanogap-based sensors. Critically, the resulting electrodes maintained their stability over the course of six months at room temperature.

## Graphical abstract

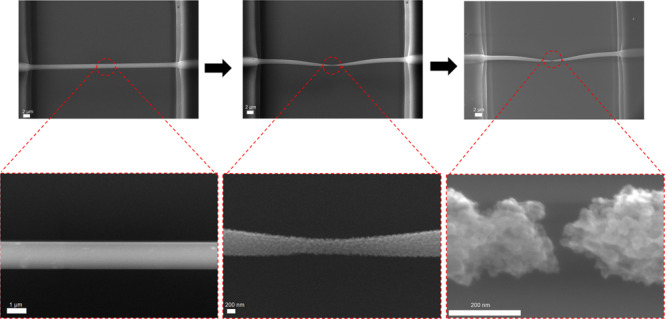

## Introduction

One of the many challenges toward the development of molecular-scale sensing devices is the issue of how to connect molecules or similar sized nanostructures to the outside world. To this end, electrodes separated by a few nanometers, known as nanogap electrodes, are commonly used to probe the electrical properties of these nanoscale objects. Consequently, the fabrication of nanogap electrodes has been the subject of great interest for researchers in the field of molecular-scale devices who employ techniques such as mechanical break junction (MBJ)^1^, focused ion beam (FIB)^2^, electromigration^3^ and photolithography^4,5^. Usually, the choice of fabrication technique is made according to the type of nanogap geometry required for the intended application. For example, MBJ or electromigration is suitable for obtaining fine point-like electrode tips, with surface areas appropriate for direct interfacing at the molecular-scale. Photolithography which can be used for the fabrication of larg rectangular nanogap electrodes whose surface area is increased to improve their performance in electrochemical sensing applications^4^. In all of these fabrication methods, the most frequently used material has been gold^6–8^. As a result of numerous studies performed using this metal, the technology is now at a point where nanogaps of only a few nanometers can be produced with yields close to 100%. However, some drawbacks from the use of this metal for the construction of nanogaps have been reported. Among the drawbacks of this type of electrode is stability; some studies have reported that after a few days at room temperature, gold nanogaps experience a widening of several nanometers, which was attributed to the surface energy and high-mobility of the material^3,9,10^. To solve this issue, different materials, such as platinum, which is a more stable metal than gold, have been used for nanogap electrodes^9,11^.

A different approach to using metals such as gold or platinum for nanogap electrodes is to explore nanostructured carbon as the material which can present advantages in terms of resistance to electromigration, better stability at or above room temperature, and easier binding to a greater variety of molecules for biosensing applications^10,12^. Fabricating carbon-based nanogap devices for molecular-scale sensing applications is usually accomplished by temperature-activated electrical breakdown of carbon nanotubes (CNTs), graphene sheets, or carbon nanofibers (CNFs)^13–15^. In numerous studies, the breakdown mechanism in these carbon devices was found to be related to both the electron transport in the sample and the experimental conditions^13,16–19^.

Because CNTs and graphene exhibit higher electrical and thermal conductivities than that exhibited by the more disordered glassy carbon nanofibers (GCNFs) obtained by the pyrolysis of some organic precursors, the former two have received considerably more attention and have been subjected to a greater number of studies detailing their breakdown process. For instance, Collins et al.^13^ reported that under a high electrical bias, the power that multiwalled CNTs are able to withstand before breakdown depends on the presence of oxygen (in air). It was concluded that, in air, the power is limited by oxidation, whereas in vacuum, the power is limited only by the current-carrying capacity of the CNTs, which can withstand a higher power level before failure. Wei et al.^20^ studied the breakdown of CNT bundles induced by Joule heating under vacuum, and this breakdown was attributed to the sublimation/evaporation of carbon at high temperatures (>3000 K). Interestingly, the authors reported that high-temperature annealing of the CNTs before breakdown led to an increase in electrical conductivity. Recently, Otsuka et al.^19^, in a study on the field emission in nanogaps fabricated from single-walled CNTs, reported nanogap sizes of less than 100 nm when performing experiments in a dry-oxygen environment. However, experiments conducted in ambient air and wet-O_2_ conditions (relative humidity: 30–100%) resulted in much larger gaps (>500 nm). The presence of water vapor was found to play a major role in post-breakdown etching, which afforded electrodes with larger nanogaps. In addition, Marquardt et al.^18^ studied the electrical breakdown of single CNTs under controlled environments of high vacuum, argon, air, and O_2_. High vacuum was the best condition for obtaining sub-10 nm nanogaps. Graphene deposited between two electrodes can be broken down under the influence of voltage. During the breakdown process, which is achieved by applying a feedback-controlled voltage under high vacuum^14^, a bowtie-like nanoconstriction structure is formed, enhancing the on/off ratio when used as a field-effect transistor. Nanoconstrictions in graphene have been the subject of many studies regarding their electric transport properties and the observation of ballistic quantum conduction^21–23^.

While the breakdown in CNTs and graphene has been extensively reported, fewer studies exist on the more disordered GCNFs^15,24–26^. A common method for the synthesis of carbon nanofibers is to derive them from a polymer by a two-part process consisting of: (1) electrospinning, to produce the polymeric nanofibers^27,28^ and (2) pyrolysis, where the nanofibers are carbonized, provided that an appropriate carbon polymeric precursor, such as polyacrylonitrile (PAN) or the epoxy-based negative photoresist SU-8, is selected^29–31^. The question of how the polymer carbon source affects the final structure of the carbon nanofibers has been of great interest for researchers and continues to this day. In general, polymer-derived carbon is classified as either graphitizing or non-graphitizing, depending on the capability of the material to be transformed into graphite above a certain temperature^32^. Different authors have proposed how the graphitizing nature of carbon is related to the arrangement of the polymer chains and the conditions at which cross-linking occurs in the precursor, which may lead to the formation of fullerene-like structures that prevent the graphitization of the material even at very high-temperatures^32,33^. For the particular case of the carbonization of SU-8, which has a composition that consists of a bisphenol A novolak resin dissolved in cyclopentanone and up to 10 wt% triarylsulfonium hexafluoroantimonate salt as a photoacid generator^34^, studies indicate a resulting non-graphitizing allotrope of carbon known as glassy carbon, which has particularly good resistance to chemical attacks, thermal stability, and biocompatibility and has been widely used in electrochemical applications^25,35,36^. Therefore, while GCNFs exhibit lower electrical and thermal conductivities than those of CNTs and graphene, the simplicity in production and the above mentioned characteristics of glassy carbon, make them an attractive alternative for the fabrication of stable carbon-based nanodevices^35,36^.

In studies on the electrical breakdown of carbon nanofibers, the phenomenon is also attributed to the high temperatures reached in the wires, the pressure, and the O_2_ levels during the experiment^15,26,37^. Previously, our group reported on the fabrication of electrospun-suspended GCNF devices and the correlation between the electrical and thermal conductivities and the volumetric changes that occur during the carbonization process^38,39^. We also studied nanogap formation on these electrospun GCNFs, revealing a correlation between the gap size and the length of the fiber, with shorter fibers producing smaller nanogaps^40^. Establishing a separation of 10 nm or less as an appropriate goal for applications in molecular-scale sensors, we learned from these previous results that GCNFs that are less than ~2 µm long were required. However, the limitations of photolithographic processes make the production of such short fibers very challenging.

Therefore, developing a process capable of achieving sub-10 nm GCNF nanogap devices without the need for sub-2 µm long GCNFs is highly desirable. The main challenge is to prevent an uncontrolled breakdown during the heating process by gradual thinning of the fiber, which would form smaller and smaller constrictions until the eventual breakdown of the fiber. In this work, a voltage application program based on monitoring the resistance changes that occur during the electrical stimulation of suspended GCNFs was implemented. In addition, to further reduce the size of the nanogaps, the mechanism behind the thinning and eventual breakdown of the suspended GCNFs was studied by carrying out experiments under four experimental conditions: (i) in a dry-air-filled chamber at atmospheric pressure, (ii) in a carbon dioxide (CO_2_-)-filled chamber at atmospheric pressure, (iii) at high vacuum in a chamber that was previously purged with dry-air, and (iv) at high vacuum in a chamber that was previously purged with CO_2_. Following the proposed procedure, for the case of a chamber at a high-vacuum previously purged with CO_2_, the average nanogap size was 9.8 nm for GCNFs with an average length of 52 µm, and separations as small as ~4 nm were produced. Furthermore, the devices proved to have excellent stability by showing no sign of degradation after approximately 6 months of storage at room-temperature.

## Materials and methods

### Glassy carbon device fabrication

**Fig. 1:**
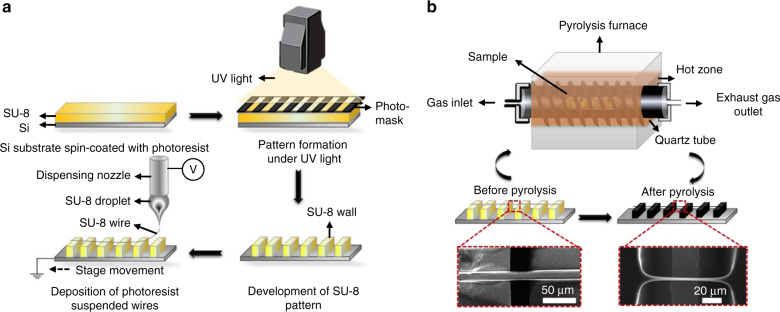
Fabrication process of the carbon-based devices. **a** Schematic representation of the photolithography fabrication procedure of the suspended GCNF device. **b** Pyrolysis was performed in a quartz tube furnace. Zoomed-in sections showing the fibers and diameter change from micro- to nano-scales before and after pyrolysis.

The first step was to deposit a fine layer of the negative photoresist SU-8 2025 (MicroChem Inc., Westborough, MA, USA) with a thickness of 20 µm on an N-type silicon wafer (100 mm) with a 1 µm thermal oxide layer (University Wafer, Boston, MA, USA) by spin coating (model WS-650–23; Laurell, North Wales, PA, USA) at a speed of 4000 rpm for 30 s. The coated surface was baked for 5 min at 95 °C on an HS61 hotplate (Torrey Pines Scientific, Inc., Carlsbad, CA, USA) to remove the solvent. Subsequently, the pattern was defined by selective exposure to ultraviolet (UV) light (2000-EC UV lamp; Dymax, Torrington, CT, USA) for 4 s. The unexposed segments were removed by submerging the wafer in a developer solution (MicroChem Inc.), leaving behind a six-wall device (Fig. 1a).

The second step was the deposition of an SU-8 2025 fiber was performed using the Electromechanical Spinning (EMS) technique in a custom-made Newport uFab electrospinning platform (Newport Corp., Irvine, CA, USA). By adjusting the needle-to-collector distance to ~1 mm and the voltage to 400 V, high control of the position of a single polymer fiber on the supporting walls was achieved. Detailed optimization of CNF fabrication using EMS was described in our previous publications^40,41^.

Finally, the complete device, including the supporting walls and suspended fibers, was placed inside a PEO 601 furnace (ATV Technologie GmbH, Vaterstetten, Germany) for pyrolysis. During this process, the structures were heated up to 900 °C in an inert N_2_ environment, resulting in the loss of noncarbon atoms of SU-8. The pyrolysis of SU-8, as previously demonstrated, yields glassy carbon structures^24,31,35^. In Fig. 1, a representation of the fabrication process of the suspended GCNF device is presented.

### Experimental chamber with controlled conditions

The experimental conditions were controlled by placing the samples in a 3 L chamber connected to a Pfeiffer HiPace 80 vacuum turbopump (Pfeiffer Vacuum, Asslar, Germany). Electrical feed-through connections were used to connect the carbon device to the voltage supply. Gas input lines were used to fill the chamber with dry-air and CO_2_. The experiments were performed under four different conditions: (i) in a dry-air-filled chamber at atmospheric pressure, (ii) in a CO_2_-filled chamber at atmospheric pressure, (iii) in high vacuum in a chamber that was previously purged with dry-air, and (iv) in high vacuum in a previously CO_2_-purged chamber. Purging cycles with dry-air and CO_2_ were performed by filling the chamber with the gas and pumping to a vacuum of 1 × 10^−4^ mbar. The process was repeated twice. A value of 2 × 10^−5^ mbar was established for all experiments performed under high-vacuum conditions.

### SEM characterization

The samples were mounted on a double-sided carbon tape and analyzed using a field emission scanning electron microscope (FE-SEM) model Nova NanoSEM 200 (FEI, Thermo Fisher Scientific, OR, USA), using an acceleration voltage of 15 kV under high vacuum with a TLD detector. The samples were also analyzed using an SEM EVO MA25 (Zeiss, Germany).

### TEM characterization

Transmission electron microscopy (TEM) experiments were performed on an FEI/Philips CM-20 conventional TEM. The samples were prepared by electrospinning directly on a silicon TEM grid with a 50 nm SiN support film and two apertures of 1.5 mm length and 100 µm width.

## Results and discussion

### Electrical stimulation of GCNFs for nanogap formation

**Fig. 2:**
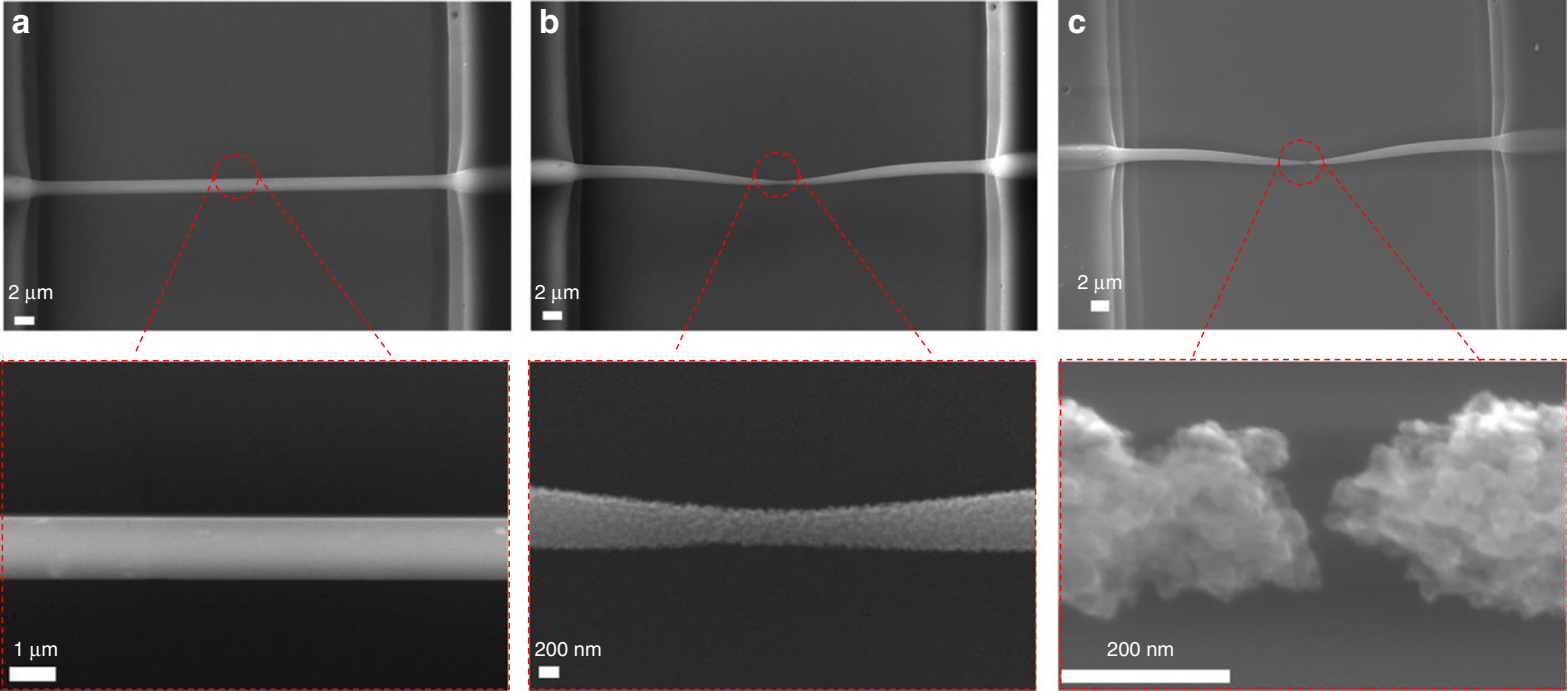
Thinning and breakdown of a GCNF due to the voltage application procedure. SEM images of **a** The original GCNF with *L* = 44.5 µm and *D* = 1.4 µm. **b** Constriction of 351 nm after the first voltage application. **c** Nanogap of 20 nm produced when the voltage was applied until an open circuit was detected.

The nanogap formation was performed using a voltage application program running a feedback-controlled loop and monitoring the electrical resistance changes (Δ*R*) of the fiber. Although no direct temperature measurements were performed on the GCNF, an indication of the effects caused by heating of the GCNF can be inferred from this Δ*R* value when the fiber is electrically stimulated. The Δ*R* parameter was therefore selected as the main indicator of changes occurring in the GCNF. A negative Δ*R* indicates a decrease in the electrical resistance as the fiber is heated, as expected from the negative coefficient of resistance of carbon. A positive Δ*R* represents the loss of mass that leads to the thinning and eventual breakdown of the GCNF. The voltage application process started by applying a low voltage (0.2 V) and the resistance was measured. The voltage was subsequently increased in steps of 0.1–0.2 V until a threshold Δ*R* value was observed, above which a reduction in the cross-sectional area of the fiber began to occur. To avoid an uncontrolled breakdown process, abrupt changes in Δ*R* must be prevented. From our experiments, we found that a positive electrical resistance change larger than Δ*R* = 0.05% is suitable to allow the system to reach the activation temperature needed to form a constriction, while preventing the breakdown process from taking place in an uncontrolled manner. An additional measure to prevent the uncontrolled breakdown of the CNFs was implemented by limiting the value at which the voltage could be increased (*V*_max_). Before the start of the program, an initial *V*_max_ was selected based on previous experiments on fibers of similar diameters and lengths. The voltage application program automatically adjusted the time at each voltage step between 1 and 4 s to further control the energy imparted on the fiber. Once Δ*R* exceeded the threshold value, the electrical stimulation was interrupted, and a new voltage application cycle started. The process was repeated until an open circuit was detected, which was indicative of the formation of a nanogap. Based on the assumption that as the CNFs were gradually thinned down, the condition at which Δ*R* *>* 0.05% was reached occurred at lower threshold voltages (*V*_th_), the maximum voltage was decreased at each voltage application cycle as *V*_max_ = *V*_th_ −0.2 to follow this tread. Thus, at any given point during the experiment, the thinned fiber that in a previous iteration caused the condition Δ*R* > 0.05% to be met was not allowed to be taken to a voltage. Figure 2 illustrates the thinning and breakdown process of a GCNF, where a fiber of 44.5 µm length and 1.4 µm diameter (Fig. 2a) was thinned down to a nanoconstriction of 351 nm (Fig. 2b). The voltage application was then allowed to run until an open circuit was detected, with the concomitant production of a 20 nm nanogap (Fig. 2c).

In addition to the voltage application, another important factor for the fabrication of sub-10 nm nanogaps is the choice of the experimental conditions for the breakdown of the fiber. According to previous reports^13,19^ and as will be explained in detail in the next sections, carbon can lose mass upon heating by two main mechanisms: (i) an oxidation reaction with O_2_ or CO_2_ and (ii) sublimation (solid to gas) at high temperatures, estimated to be approximately 4000 K at atmospheric pressure and 3000 K in high vacuum^42^. The following sections describe experiments performed under atmospheric and high-vacuum conditions.

### Nanogap formation under atmospheric pressure

**Fig. 3:**
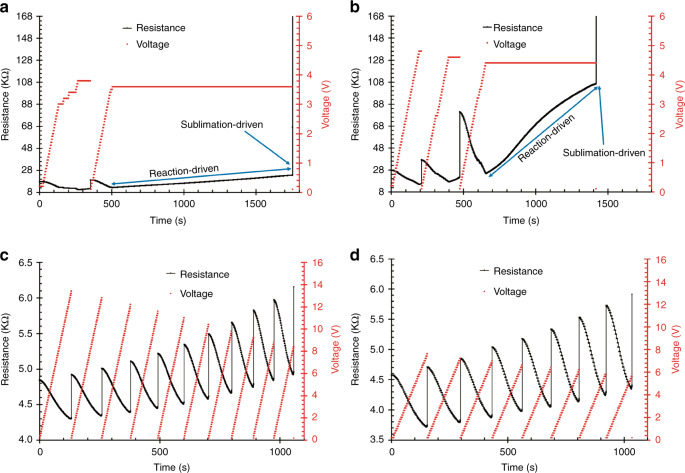
Resistance evolution for the thinning and breakdown process for: **a** dry-air at atmospheric pressure (GCNF with *L* = 41.5 µm and *D* = 1.1 µm), **b** CO_2_ at atmospheric pressure (GCNF with *L* = 39.4 µm and *D* = 1.0 µm), **c** high vacuum after purging the chamber with dry-air (GCNF with *L* = 48.7 µm and *D* = 1.6 µm), and **d** high vacuum after purging the chamber with CO_2_ (GCNF with *L* = 57.2 µm and *D* = 1.2 µm).

The nanogap formation mechanism was first studied in a chamber at atmospheric pressure filled with dry-air using the above-described voltage application procedure. Figure 3a shows the resistance evolution for the GCNF thinning process under these conditions. The voltage was stepped up from 0.2 to *V*_max_ = 3.8 V, with a step size of 0.2 V, and the resistance first decreased from 18 to 10 kΩ and then increased at the end of this voltage range. Once the condition Δ*R* >0.05% was detected, the voltage application was interrupted, *V*_max_ was decreased to 3.6 V, and the process was repeated. From 500 to 1750 s, a slow and gradual increase in the electrical resistance was recorded at 3.6 V, followed by an abrupt increase in resistance at the end of this period. This performance was always observed for experiments in air at atmospheric pressure and could be explained in terms of the two main factors that consume the GCNF: oxidation and sublimation. Oxidation, which is due to the presence of O_2_ in the chamber, is an expected process because the autoignition temperature of carbon is ~973 K. This observation suggests that, even at temperatures lower than the sublimation temperature of carbon, the fiber could be thinned down by burning. When the temperature reaches the sublimation temperature of carbon, which is estimated to be approximately 4000 K under atmospheric pressure, the thinning mechanism switches from reaction-driven to sublimation-driven, producing a rapid and abrupt jump in the electrical resistance, which hinders the control of the breakdown. Using the above described method for ten GCNFs of an average length of 43.3 µm under these conditions, nanogaps with an average size of 154 nm with a standard deviation of 43.3 nm were obtained. Fig. S1 in the supplementary information shows the SEM images of some GCNFs broken under these conditions.

To avoid reactions due to the presence of O_2_ in the experimental setup, tests were also performed at atmospheric pressure in a chamber filled with CO_2_ (see the experimental section). Figure 3b shows a typical plot for a GCNF treated under these conditions. With initial *V*_max_ = 4.8 V, from 0 to 500 s, two cycles of voltage application were performed, to avoid the rapid breakdown of the fiber. Then, at approximately 650 s, with the maximum voltage set at 4.4 V, a much faster electrical resistance increase than that in Fig. 3a was observed. In this case, the reactions were promoted not by the presence of O_2_ but by CO_2_. From the comparison of the resistance slopes of Fig. 3a, b, it can be immediately inferred that the fiber was consumed at a higher rate in CO_2_ than in air. This is most likely because the reaction in the CO_2_-filled chamber (100% CO_2_ content) (i.e., C + CO_2_ ⇒ 2CO) was thermodynamically favored at temperatures above ~1600 K and the reaction rate was high compared with the reaction rate in air (21% O_2_ content) (i.e., 2C + O_2_ ⇒ 2CO)^43,44^. Similar to the case of the reaction in air, a sudden and abrupt increase in the electrical resistance at 1400 s was observed, indicating that sublimation was activated due to the high temperature, which resulted in the breaking of the fiber. Ten GCNFs with an average length of 50 µm were thinned and broken, resulting in gaps with an average size of ~144 nm and a standard deviation of 13.5 nm. Fig. S2 shows the SEM images of some of the GCNFs broken in a CO_2_ filled chamber at atmospheric pressure.

### Nanogap formation in high vacuum

Under atmospheric pressure in either an air-filled or a CO_2_-filled chamber, the oxidation reactions during the experiment contribute significantly to the unpredictability/uncontrollability of the breakdown process and thus larger nanogaps. Furthermore, at atmospheric pressure, sublimation is expected to occur at the extremely high temperature of ~4000 K. With the aim of further reducing the nanogap size, we used the voltage application method for samples in high vacuum (2 × 10^−5^ mbar). Figure 3c shows a typical plot of the applied voltage and the resistance evolution of a GCNF during voltage stimulation. As observed, the process ran for 10 cycles without experiencing the unpredictability that was observed at atmospheric pressure. By performing the experiments under vacuum conditions, ten fibers 45 μm in length with an average nanogap size of 102 nm and a standard deviation of 52.7 nm were obtained. Fig. S3 shows the SEM images of several of the GCNFs broken under these conditions. In this scenario, fiber thinning is mainly related to sublimation, which facilitates the control of the process. In contrast, the thinning/breakdown of the GCNF under atmospheric pressure is dependent on the reactions of C with O_2_ or CO_2_, as well as on the sublimation process. Upon approaching fiber breakdown, these oxidation reactions speed up the breakdown process, whereas in the absence of reactive gases such as O_2_ or CO_2_, only sublimation is expected to influence the breakdown process. Thus, the voltage application method exhibits excellent performance under high-vacuum conditions, resulting in small nanogaps. Moreover, the sublimation of GCNFs could occur at lower temperatures (~3000 K) under these conditions.

**Fig. 4:**
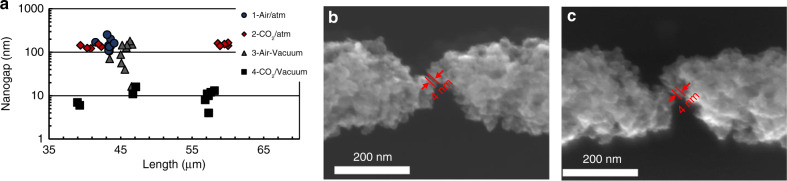
Resulting nanogaps after following the voltage application procedure under four different environments. **a** Measured nanogaps for the four different environments tested. **b** SEM image showing the ~4 nm nanogap produced under high vacuum after purging the chamber with CO_2_. The GCNF was 57.5 µm in length, with an initial diameter of 1.2 µm. **c** SEM image of the nanogap shown in **b** taken after 6 months of storage at room temperature. There is no apparent change in the separation between the electrodes.

When reaching even smaller nanogap sizes, one can expect that as the constrictions in the GCNF become thinner, the process becomes more difficult to control. As discussed in the previous sections, the presence of O_2_ or CO_2_ can lead to reactions that promote the burning of the GCNF. In high vacuum of ~2 × 10^−5^ mbar, although the rate of the oxidation reactions is reduced, there might still be traces of CO_2_ and O_2_, which can have an effect on the nanowire thinning/breakdown mechanism. To remove any remnant of oxygen, the chamber was purged with CO_2_ before the high-vacuum experiments were performed. As shown in Figs. 3c, d, the resistance response was similar in both high-vacuum processes (with or without CO_2_ purging). However, the nanogaps produced after the chamber was purged with CO_2_ were significantly smaller, with an average size of 9.8 nm and a standard deviation of 7.4 nm for ten GCNFs with an average length of 52 µm. Considering a stringent 10 nm or less gap as the target separation, a yield of 50% was achieved for the ten CNFs broken under high vacuum after CO_2_ purge; however, all fibers were below 20 nm. This reduction in the nanogap size can be observed in Fig. 4a, where the measured data points for all four environments are shown. A minimum nanogap size of ~4 nm was achieved in these experiments, as shown in the SEM image in Fig. 4b. The stability of the carbon nanogaps was tested by performing SEM analysis of several samples after they were stored at room-temperature for approximately 6 months. The new SEM images show no change in shape or nanogap separation, demonstrating the good stability of these structures. Figure 4c shows the SEM image of the ~4 nm nanogaps after 6 months. Fig. S4 shows the SEM images of several of the sub-10 nm nanogaps broken under these conditions.

From these results, it can be concluded that the use of the reported voltage application procedure under high vacuum in a chamber that was previously purged with CO_2_ afforded a good approach for the fabrication of sub-10 nm nanogaps on suspended GCNFs without the need of fibers that are sub-2 µm long. As in the case of high-vacuum conditions, the sublimation temperature for a chamber filled with air is expected to be lower than that for a chamber at atmospheric pressure (~3000 K), and the process would be driven mostly by the sublimation of the fiber. Further reduction in the nanogap size may be accomplished considering the traces of particles that remain in the chamber after vacuum conditions are reached. In the case of air, the presence of small quantities of O_2_ may lead to exothermic reactions that release energy into the system, causing the fiber to continue burning even after the electrical stimulation is stopped. In contrast, for CO_2_, the remaining particles undergo an endothermic reaction that consumes energy from the system, thereby contributing to a better control over the breakdown of the fiber, with the concomitant production of sub-10 nm nanogaps.

### Surface and structural effects on the GCNFs

**Fig. 5:**
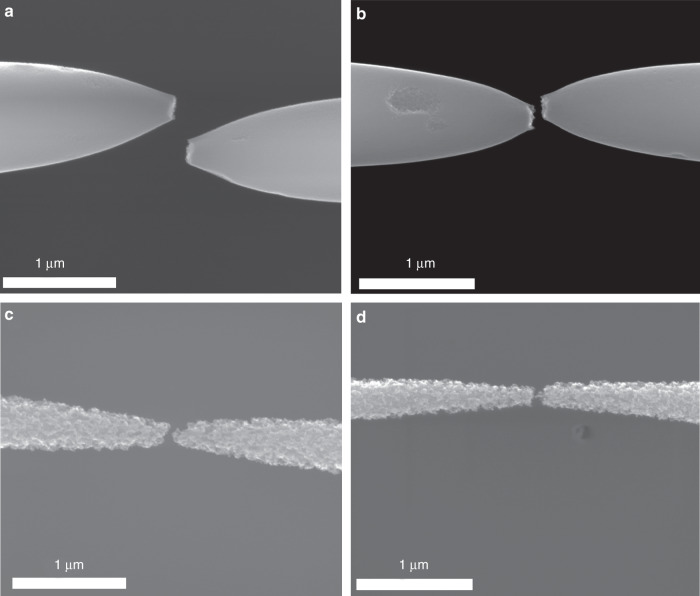
Different morphologies of the resulting nanogap electrodes under the four different environments tested. SEM images showing the smooth and rough GCNF surfaces broken under **a** dry-air at atmospheric pressure, **b** CO_2_ at atmospheric pressure, **c** high vacuum in a chamber that was previously purged with dry-air, and **d** high vacuum in a chamber that was purged with CO_2_.

In this section, we analyze the surface of the resulting GCNFs after the thinning and breakdown processes. In Fig. 5, the SEM images of four different fibers are presented. It can be clearly observed that the experiments performed under an atmospheric pressure of air and CO_2_ (Fig. 5a, b, respectively) resulted in a smooth, almost polished, surface. This effect can be attributed to the reactions that drive the burning of the fiber prior to sublimation, because the oxidation of fibers in air and even in CO_2_ has been previously reported and used to treat the surface of CNFs^45,46^. On the other hand, in the high-vacuum cases, the sublimation-driven process can be envisaged as responsible for the breaking of the bonds in the disordered GCNF, leading to the very rough surfaces observed in Fig. 5c, d.

**Fig. 6:**
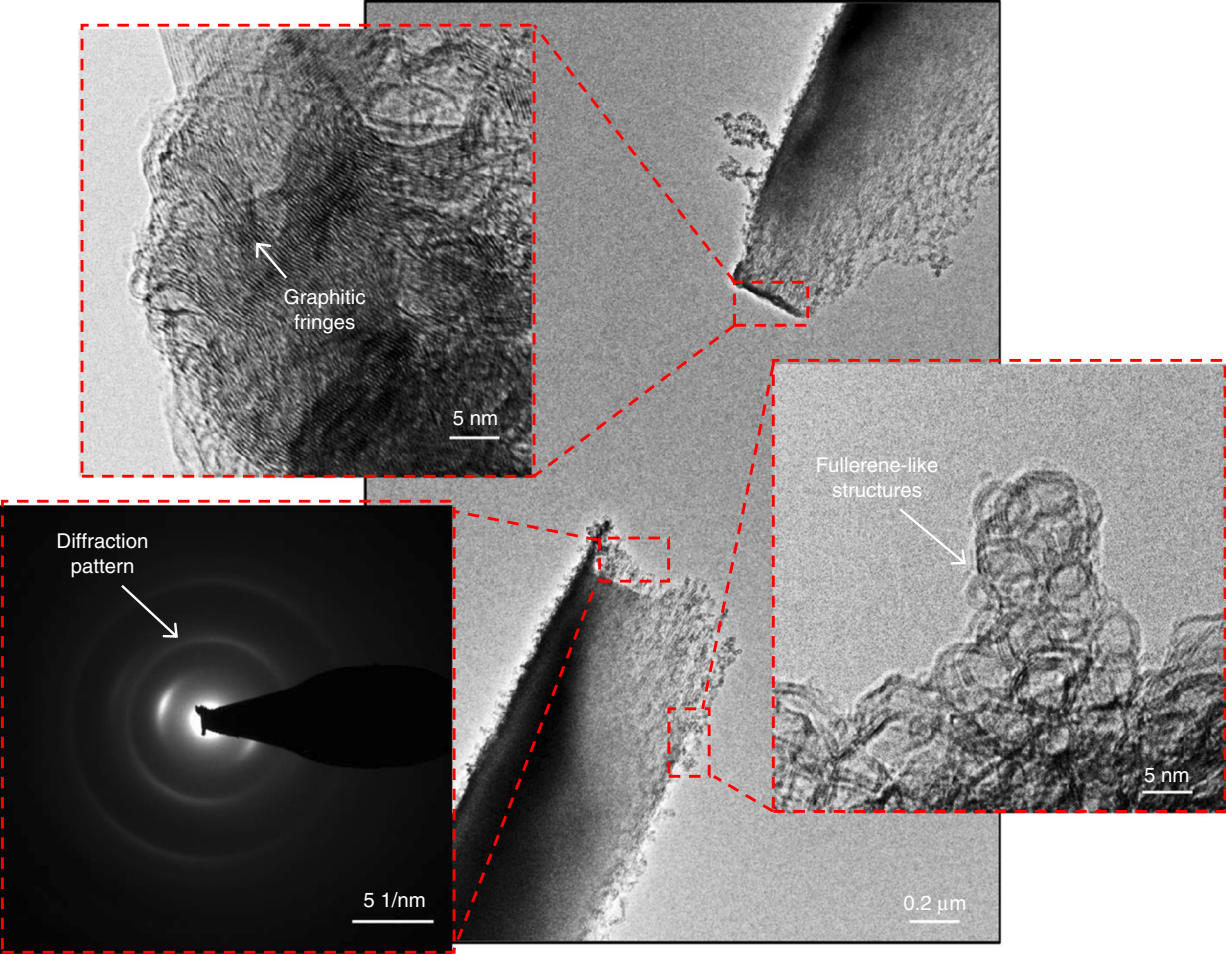
TEM images of a nanogap obtained under high-vacuum conditions. The magnified images show the fullerene-like rings present at the edge of the fiber, as well as the graphitic fringes and the diffraction pattern on the tip of the electrode.

Finally, the effect of the high temperatures reached in the GCNF during the thinning process was studied by TEM. Figure 6 displays the TEM image of fullerene-like rings at the edges of a broken fiber. The presence of these fullerene structures on carbons obtained through the pyrolysis of organic precursors has been associated with the bending of the polymeric chains during cross-linking in the fabrication process^33^. Furthermore, given the stability of fullerenes at high temperatures, their presence in non-graphitizing carbons has been proposed as the main factor responsible for preventing graphitization^33,47^. Interestingly, at the very tip of the broken electrodes, the region where the maximum temperature was expected to occur, we observed high concentration of graphitic fringes, which, when considered with the bright zones observed in the rings of the diffraction pattern, indicates the presence of a certain order at the breakdown point. Rearrangement of the structure of non-graphitizing glassy carbon, resulting in a greater degree of order, was recently reported for a sample that was subjected to a very high compression pressure (45 GPa) and was attributed to the destruction of the fullerene bonds that prevented graphitization^48^. Although further studies are required to explain the apparent reordering of glassy carbon that occurs during the nanogap formation process, this initial result indicates that the decomposition of the GCNF bonds at extremely high temperatures may indeed result in a more extensive graphitic region at the very tip of the electrode.

## Conclusion

With the goal of using the Joule-heating induced breakdown of GCNFs to fabricate ~10 nm nanogap electrodes, suitable for molecular-scale sensing applications, without the need to use fibers with lengths on the order of 2 µm or less, we studied a nanogap fabrication strategy in which a feedback loop was used to thin down and break the fibers under four different environmental conditions. The stimulation voltage was manipulated as a function of the change in fiber resistance. In addition, we studied the impact of the fiber thinning conditions on the resulting gaps. Thus, for fibers thinned at atmospheric pressure in the presence of O_2_ or CO_2_, gaps above 130 nm were created by the reaction-driven burning of the fiber at a high sublimation temperature. However, in the experiments performed at high vacuum, the main thinning/breakdown mechanism was the sublimation of carbon, which was reached at a lower temperature than that reached under atmospheric pressure. Under these conditions, the nanogap formation was better controlled. Finally, the presence of traces of O_2_ was minimized by purging the chamber with CO_2_ before creating the high vacuum, which allowed the fabrication of nanogaps with an average size of 9.8 nm in fibers with a length of 52 µm, with a minimum recorded gap of ~4 nm. The SEM images of the carbon nanogap electrodes, after being stored for 6 months at room temperature, show no apparent change in nanogap size or electrode shape. This observation proves the great stability of CNFs as an electrode material. Our strategy offers a relatively simple method for sub-10 nm nanogap fabrication using GCNFs with lengths of a few tens of micrometers, which could open a window of opportunity for the fabrication of nanoscale devices for biosensing applications. Finally, as the graphitic regions in the TEM images indicate, an additional benefit of our nanogap fabrication method, is that it facilitates the study of the transformation of disordered carbons, acting as nanofurnace platforms that allow for the study of the structure of carbon at extreme temperatures, given the difficulty of using furnaces that can reach temperatures above 3000 K.

## Supplementary information

Supplementary information.

## References

[1] Xiang D, Jeong H, Lee T, Mayer D (2013). Mechanically controllable break junctions for molecular electronics. Adv. Mater..

[2] Nagase T, Kubota T, Mashiko S (2003). Fabrication of nano-gap electrodes for measuring electrical properties of organic molecules using a focused ion beam. Thin Solid Films.

[3] Strachan DR (2006). Clean electromigrated nanogaps imaged by transmission electron microscopy. Nano Lett..

[4] Hammond J, Rosamond M, Sivaraya S, Marken F, Estrela P (2016). Fabrication of a horizontal and a vertical large surface area nanogap electrochemical sensor. Sensors.

[5] Hatsuki R, Yujiro F, Yamamoto T (2013). Direct measurement of electric double layer in a nanochannel by electrical impedance spectroscopy. Microfluid Nanofluid.

[6] Perrin ML (2013). Large tunable image-charge effects in single-molecule junctions. Nat. Nano..

[7] Johnston DE, Strachan DR, Johnson ATC (2007). Parallel fabrication of nanogap electrodes. Nano Lett..

[8] Liang W, Shores MP, Bockrath M, Long JR, Park H (2002). Kondo resonance in a single-molecule transistor. Nature.

[9] Prins F (2009). Room-temperature stability of Pt nanogaps formed by self-breaking. Appl. Phys. Lett..

[10] Prins F (2011). Room-temperature gating of molecular junctions using few-layer graphene nanogap electrodes. Nano Lett..

[11] Kervennic YV, Van der Zant HSJ, Morpurgo AF, Gurevich L, Kouwenhoven LP (2002). Nanometer-spaced electrodes with calibrated separation. Appl. Phys. Lett..

[12] Yan H, Bergren AJ, McCreery RL (2011). All-carbon molecular tunnel junctions. J. Am. Chem. Soc..

[13] Collins PG, Hersam M, Arnold M, Martel R, Avouris P (2001). Current saturation and electrical breakdown in multiwalled carbon nanotubes. Phys. Rev. Lett..

[14] Lu Ye (2010). High-On/Off-ratio graphene nanoconstriction field-effect transistor. Small.

[15] Suzuki M (2007). Current-induced breakdown of carbon nanofibers. J. Appl. Phys..

[16] Qi P (2004). Miniature organic transistors with carbon nanotubes as quasi-one-dimensional electrodes. J. Am. Chem. Soc..

[17] Pop E, Mann DA, Goodson KE, Dai H (2007). Electrical and thermal transport in metallic single-wall carbon nanotubes on insulating substrates. J. Appl. Phys..

[18] Marquardt CW (2010). Electroluminescence from a single nanotube-molecule-nanotube junction. Nat. Nano..

[19] Otsuka K, Inoue T, Shimomura Y, Chiashi S, Maruyama S (2016). Field emission and anode etching during formation of length-controlled nanogaps in electrical breakdown of horizontally aligned single-walled carbon nanotubes. Nanoscale.

[20] Wei, Y, Jiang, Liu, L, Chen, Z. & Fan, S. Vacuum-breakdown-induced needle-shaped ends of multiwalled carbon nanotube yarns and their field emission applications*.**Nano Lett.* **7**, 3792–3797 (2007).

[21] Tombros N (2011). Quantized conductance of a suspended graphene nanoconstriction. Nat. Phys..

[22] Terrés B (2016). Size quantization of Dirac fermions in graphene constrictions. Nat. Commun..

[23] Gehring P (2016). Quantum interference in graphene nanoconstrictions. Nano Lett..

[24] Park BY, Taherabadi L, Wang C, Zoval J, Madou MJ (2005). Electrical properties and shrinkage of carbonized photoresist films and the implications for carbon microelectromechanical systems devices in conductive media. J. Electrochem. Soc..

[25] Lentz CM, Samuel BA, Foley HC, Haque MA (2010). Synthesis and characterization of glassy carbon nanowires. J. Nanomater..

[26] Maeda S, Wilhite P, Kanzaki N, Yamada T, Yang CY (2011). Change in carbon nanofiber resistance from ambient to vacuum. AIP Adv..

[27] Reneker DH, Chun I (1996). Nanometre diameter fibres of polymer, produced by electrospinning. Nanotechnology.

[28] Sun D, Chang C, Li S, Lin L (2006). Near-field electrospinning. Nano Lett..

[29] Inagaki M, Yang Y, Kang F (2012). Carbon nanofibers prepared via electrospinning. Adv. Mater..

[30] Newcomb BA (2016). Processing, structure, and properties of carbon fibers. Compos Part A.

[31] Sharma CS, Sharma A, Madou MJ (2010). Multiscale carbon structures fabricated by direct micropatterning of electrospun mats of SU-8 photoresist nanofibers. Langmuir.

[32] Burket CL, Rajagopalan R, Foley HC (2008). Overcoming the barrier to graphitization in a polymer-derived nanoporous carbon. Carbon.

[33] Ghazinejad M, Holmberg S, Pilloni O, Oropeza-Ramos L, Madou M (2017). Graphitizing non-graphitizable carbons by stress-induced routes. Sci. Rep..

[34] del Campo A, Greiner C (2007). SU-8: a photoresist for high-aspect-ratio and 3D submicron lithography. J. Micromech. Microeng..

[35] Mardegan A (2013). Optimization of carbon electrodes derived from epoxy-based photoresist. J. Electrochem Soc..

[36] Wang C, Madou M (2005). From MEMS to NEMS with carbon. Biosens. Bioelectron..

[37] Kitsuki, H. et al. Current-induced breakdown of carbon nanofibers for interconnect applications. In *7th IEEE Conference on Nanotechnology* IEEE-NANO 2007 342–345 (2007).

[38] Ferrer-Argemi L (2018). Size-dependent electrical and thermal conductivities of electro-mechanically-spun glassy carbon wires. Carbon.

[39] Canton G, Do T, Kulinsky L, Madou M (2014). Improved conductivity of suspended carbon fibers through integration of C-MEMS and Electro-Mechanical Spinning technologies. Carbon.

[40] Salazar A, Cardenas-Benitez B, Pramanick B, Madou MJ, Martinez-Chapa SO (2017). Nanogap fabrication by Joule heating of electromechanically spun suspended carbon nanofibers. Carbon.

[41] Bisht GS (2011). Controlled continuous patterning of polymeric nanofibers on three-dimensional substrates using low-voltage near-field electrospinning. Nano Lett..

[42] Begtrup GE (2007). Probing nanoscale solids at thermal extremes. Phys. Rev. Lett..

[43] Gao, X. Y., Zhang, Y. N., Li, B. X. & Dong, L. CFD modeling of an entrained flow gasifier. In *Applied Mechanics and Materials* (Trans Tech Publications Ltd., Switzerland, 2014).

[44] Xie J, Zhong W, Jin B, Shao Y, Liu H (2012). Simulation on gasification of forestry residues in fluidized beds by Eulerian–Lagrangian approach. Bioresour. Technol..

[45] Ehrburger, P. Surface properties of carbon fibres. In *Carbon Fibers Filaments and Composites* 147–161 (Springer, Dordrecht, 1990).

[46] Finegan IC, Tibbetts GG, Glasgow DG, Ting J-M, Lake ML (2003). Surface treatments for improving the mechanical properties of carbon nanofiber/thermoplastic composites. J. Mater. Sci..

[47] Harris PJF (1997). Structure of non-graphitising carbons. Int. Mater. Rev..

[48] Shiell TB (2018). Graphitization of glassy carbon after compression at room temperature. Phys. Rev. Lett..

