# Supplementary material for: Sub-10 nm nanogap fabrication on suspended glassy carbon nanofibers
Source: Microsyst Nanoeng. 2020 Jan 27;6:9. doi: 10.1038/s41378-019-0120-z (PMC8433410; doi:10.1038/s41378-019-0120-z)
Supplement: Supplementary file 1 — Supplementary information. [file 41378_2019_120_MOESM1_ESM.docx]

**Supplementary Information**

**Sub-10 nm nanogap fabrication on suspended glassy carbon nanofibers**

Arnoldo Salazar, Samira Hosseini, Margarita Sanchez-Domínguez, Marc. J. Madou, Alejandro Montesinos-Castellanos, Sergio O. Martinez-Chapa

1. **Glassy carbon fibers broken under dry air at atmospheric pressure**

Fibers broken with the voltage application program to glassy carbon nanofibers (CGNFs), under a dry-air environment at atmospheric pressure resulted large nanogaps above 100 nm. For ten fibers of average length 43.3 µm, an average separation of 149.8 nm was obtained. In this case, the burning of the fiber resulted in a harder to control process. In Fig. S1, SEM micrographs of some of the nanogaps created at these conditions are shown.


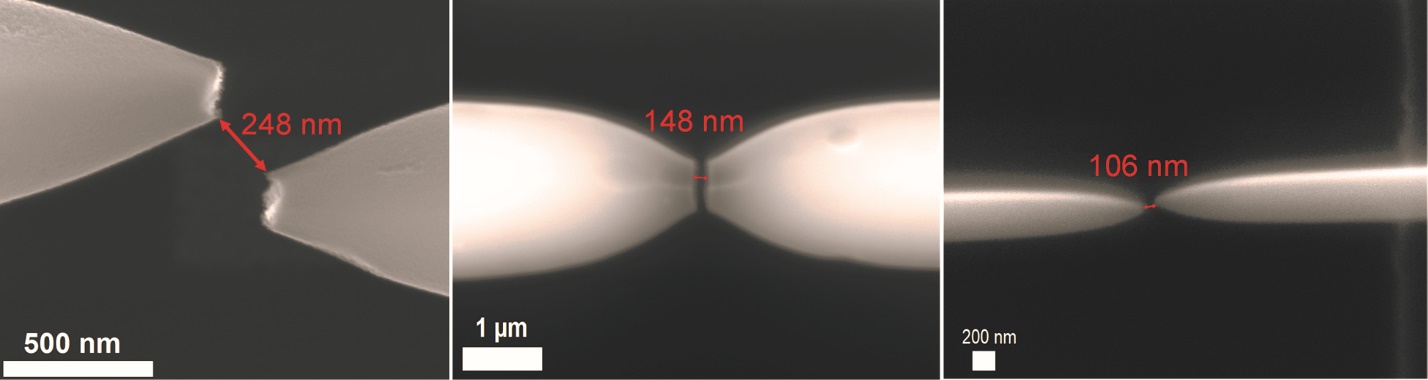


**Fig. S1** SEM micrographs of GCNFs broken with the voltage application program under dry-air at atmospheric pressure.

1. **Glassy carbon fibers broken under CO_2_ at atmospheric pressure**

To observe if the reduction of pure oxygen results in smaller nanogaps, the experimental chamber was filled with CO_2_. Using the reported voltage application procedure under these conditions resulted in nanogaps with an average separation of 152 nm for ten fibers of 50 µm in length. In Fig. S2, SEM micrographs of some of the nanogaps created at these conditions are shown.


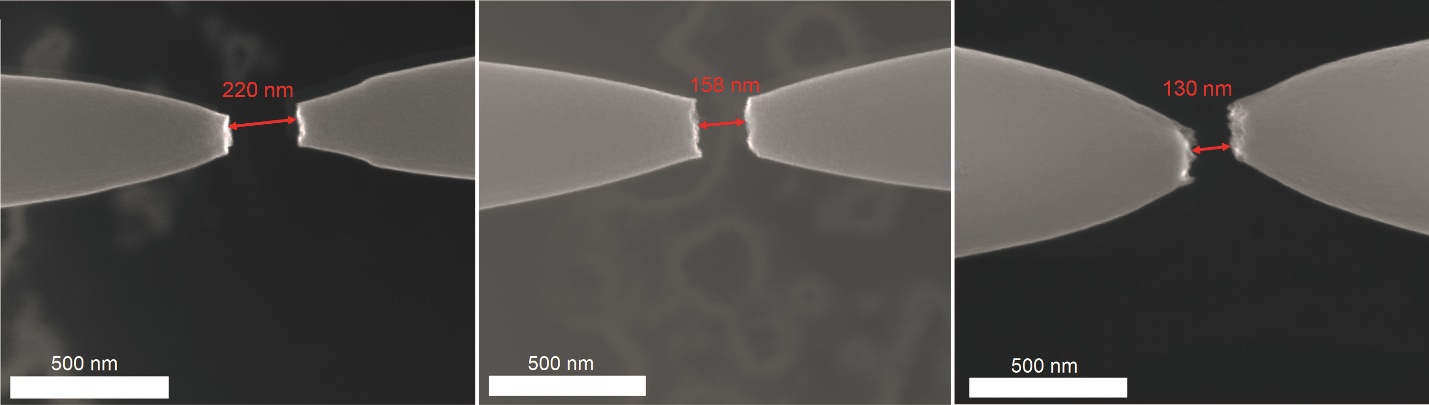


**Fig. S2** SEM micrographs of GCNFs broken with the voltage application program under CO_2_ at atmospheric pressure.

1. **Glassy carbon fibers broken under high-vacuum after purging the chamber with dry-air**

After purging the chamber with dry-air to remove humidity, the chamber was taken to a vacuum of 2 x 10^-5^ mbar. An average nanogap separation of 102 nm was obtained for fibers with average length of 45 µm. In Fig. S3, SEM micrographs of some of the nanogaps created at these conditions are shown.


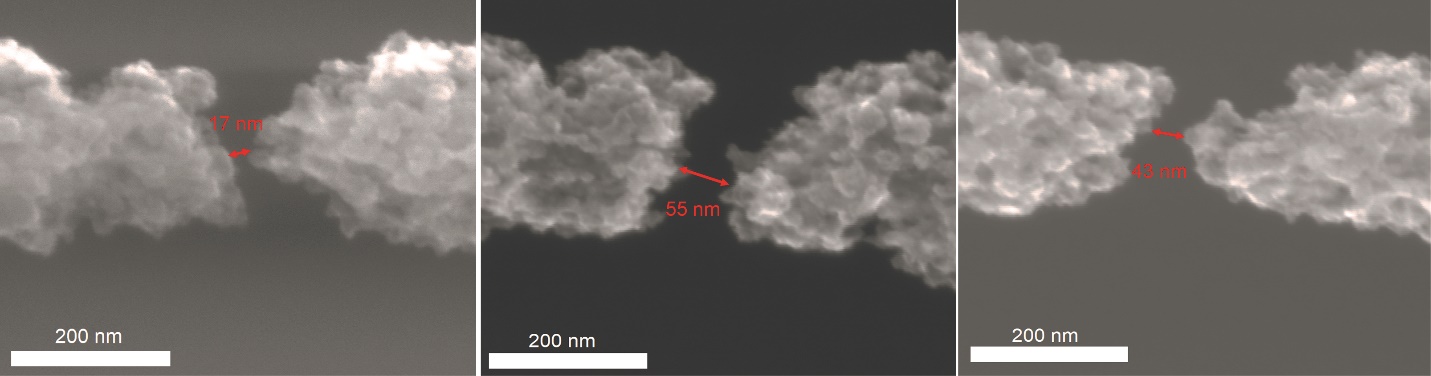


**Fig. S3** SEM micrographs of GCNFs broken with the voltage application program under a high-vacuum chamber after dry air purging.

1. **Glassy carbon fibers broken under high-vacuum after purging the chamber with CO_2_**

The final experimental conditions consisted in purging the chamber with CO_2_ to remove humidity and any remnants of O_2_, then the chamber was taken to a vacuum of 2 x 10^-5^ mbar. An average nanogap separation of 10 nm was obtained for fibers with average length of 51 µm. In Fig. S4, SEM micrographs of some of the nanogaps created at these conditions are shown.


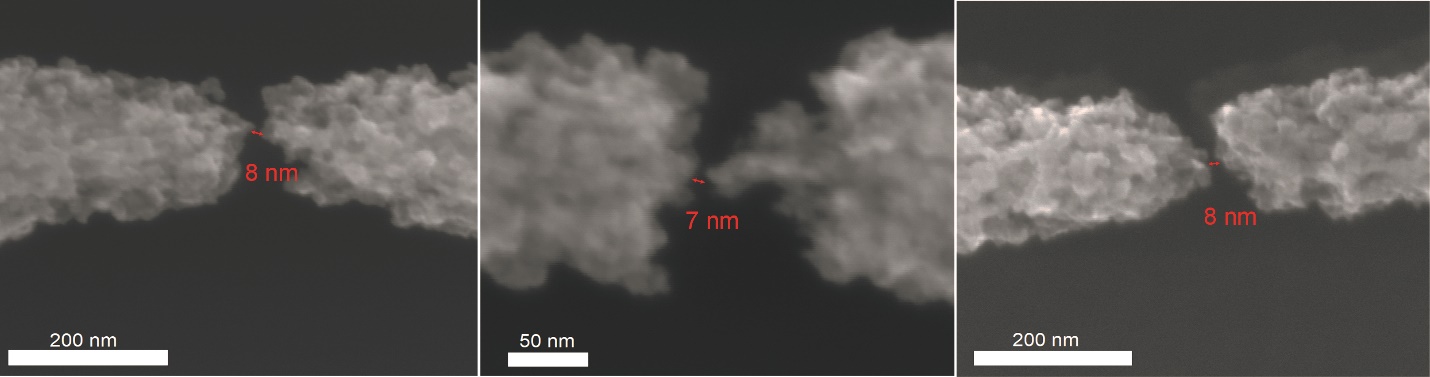


**Fig. S4** SEM micrographs of GCNFs broken with the voltage application program under a high-vacuum chamber after CO_2_ purging.
